# General Anthropometric and Specific Physical Fitness Profile of High-Level Junior Water Polo Players

**DOI:** 10.2478/v10078-012-0032-6

**Published:** 2012-05-30

**Authors:** Miran Kondrič, Ognjen Uljević, Goran Gabrilo, Dean Kontić, Damir Sekulić

**Affiliations:** 1 University of Ljubljana, Faculty of Sport, Slovenia.; 1 University of Split, Faculty of Kinesiology, Croatia.; 1 University of Dubrovnik; Waterpolo Club Jug Dubrovnik, Croatia.

**Keywords:** morphology, sport-specific test, reliability, water sports

## Abstract

The aim of this study was to investigate the status and playing position differences in anthropometric measures and specific physical fitness in high-level junior water polo players.

The sample of subjects comprised 110 water polo players (17 to 18 years of age), including one of the world’s best national junior teams for 2010. The subjects were divided according to their playing positions into: Centers (N = 16), Wings (N = 28), perimeter players (Drivers; N = 25), Points (N = 19), and Goalkeepers (N = 18). The variables included body height, body weight, body mass index, arm span, triceps- and subscapular-skinfold. Specific physical fitness tests comprised: four swimming tests, namely: 25m, 100m, 400m and a specific anaerobic 4x50m test (average result achieved in four 50m sprints with a 30 sec pause), vertical body jump (JUMP; maximal vertical jump from the water starting from a water polo defensive position) and a dynamometric power achieved in front crawl swimming (DYN).

ANOVA with post-hoc comparison revealed significant differences between positions for most of the anthropometrics, noting that the Centers were the heaviest and had the highest BMI and subscapular skinfold. The Points achieved the best results in most of the swimming capacities and JUMP test. No significant group differences were found for the 100m and 4x50m tests. The Goalkeepers achieved the lowest results for DYN.

Given the representativeness of the sample of subjects, the results of this study allow specific insights into the physical fitness and anthropometric features of high-level junior water polo players and allow coaches to design a specific training program aimed at achieving the physical fitness results presented for each playing position.

## Introduction

Water polo is an Olympic team water sport which has been played for over a century. While the rules of the game have evolved considerably over this time, physiologically speaking the sport has consistently remained a highly demanding activity (Smith, 1998). The game is oriented toward two goals positioned at either end of the swimming pool, while the playing team consists of six field players and one goalkeeper. The offensive positions include: one Center (a.k.a. two-meter offense, 2-meters, hole set, set, hole man, bucket, pit player or pit-man), two Wings (located on or near the 2-meter line), two Drivers (perimeter players, also called “flats”, located on or near the 5-meter line), and one Point (usually just behind the 5-meter line), positioned farthest from the goal. Defensive positions are often positioned the same, but just switched from offense to defense. The winner of the game is the team that scores the most goals. Game play involves swimming, treading water (using a sort of kicking motion known as an “eggbeater kick”), with players passing the ball while being defended by opponents, and scoring by throwing the ball into a net defended by a goalkeeper. Figuratively, water polo could be described as a combination of handball and swimming. While it has been hypothesized that water polo is a mixed aerobic-anaerobic activity, due to the evident problem of accurately measuring the physiological and metabolic variables during a real-game situation (i.e. in the water), empirical studies investigating physiological responses during a water polo game are scarce.

Although some papers have reported the physiological characteristics of water polo players measured through laboratory testing (Frenkl et al., 2001) and field testing procedures (Aleksandrovic et al., 2011), recent studies highlight the need for a position-specific approach to the study of water polo. For example, in a recent investigation Melchiori et al. (2010) analyzed blood lactate and game activity among elite male water polo players and found 7.7±1.0 mmol/l of blood lactate concentration, but with enormous differences among playing positions. In short, the mean match blood-lactate concentrations for Center Forwards (Centers), Center Defenders (Points), and Field Players (Drivers and Wings) were (in mmol /l) 11.2 ± 1.0, 6.7 ± 0.9, and 5.3 ± 0.9, respectively, indicating the different physiological backgrounds of the water polo game for each playing position. Authors have evidently recognized the need for a position-specific approach to the study of water polo and therefore most of the recent studies have employed such an experimental approach. Ferragut et al. (2011) investigated differences between water polo playing positions among 19 elite Spanish players in anthropometry and throwing velocity and found a higher body mass, BMI, and muscle mass of the Center Forwards compared to the Wings, and a longer foot length of the Center Backs compared with the Wings, reflecting a specific physical profile for each playing position. Very similar conclusions were made in earlier studies using similar samples of subjects (Vila et al., 2010). A similar approach (i.e. position-specific analysis) is evident when authors described the fitness and/or anthropometric characteristics of water polo athletes of both sexes (M. Lozovina et al., 2009; Tan et al., 2009), in studies which developed and validated sport-specific tests (Mujika et al., 2006; Platanou, 2005), investigations which focused on the intensity of the game (V. Lozovina, et al., 2003), or sport tactics and related statistics of the water polo game (Platanou, 2004). However, most of the studies mentioned so far sampled adult athletes (e.g. senior-age water polo players), while position specifics were mostly analyzed among three or four playing positions (i.e. goalkeepers were frequently not included in the analysis, and/or drivers and wings were observed as a single group – field players). As far as we are aware both problems are understandable. Water polo is not one of the most popular sports in the world (like football or basketball for example) and it is therefore hard to find an appropriate sample of subjects (i.e. adequate number of adequately trained athletes). This is chiefly the case with goalkeepers (one or two in each team). The second problem (e.g. studies not sampling young athletes) is also a logical consequence of the available number of subjects. Most particularly, if the study of adolescent athletes is intended then, due to the process of biological maturation, the subjects have to be near the end of puberty and homogenous in age (one or two years’ age difference at the most) and/or biological age must be controlled in the analysis (Faigenbaum, et al., 2009; Gurd and Klentrou, 2003; Latt, et al., 2009; Nindl et al., 1995). Since diversity in age is not a factor which can influence anthropometric status and/or motor achievements in adulthood (i.e. senior-age athletes), it is logically more convenient to study adult athletes.

The overall status of athletes in most sports can be observed during general and specific fitness tests. While general fitness tests (i.e. general motor and/or endurance capacities) are important indices of overall fitness status and allow a comparison of athletes from different sports (Frenkl et al., 2001), specific fitness tests allow a more precise insight into sport-specific capacities and therefore provide a basis for comparing athletes in the same sport (Bampouras and Marrin, 2009; Holloway et al., 2008; Hughes et al., 2003; Sattler et al., 2011). However, there is a clear lack of studies dealing with specific physical fitness profiles in water polo and, in particular, we found no study which has investigated this problem among high-quality junior water polo players.

The aim of this study was to investigate the status and differences between five playing positions (Goalkeepers, Centers, Drivers, Wings and Points) in anthropometric measures and some specific physical fitness variables in high-level junior (17 to 18 years of age) water polo players.

## Material and Methods

### Participants

The sample of subjects consisted of a total of 110 high-level water polo junior players. All subjects were 17 to 18 years of age and the sample of subjects included members of one of the world’s best national junior teams in 2010. All players had been trained in water polo for at least 7 years. They have trained at least 6 days per week, while each training session lasted about 2 hours. During summer period (three months) they mostly participate at two training sessions per day. Morning training is usually consisted of swimming, gym and technical training, while tactical training is done afternoon. The subjects were divided according to their playing positions into: Centers (N = 16), Wings (N = 28), perimeter players (Drivers; N = 25), Points (N = 19), and Goalkeepers (N = 18).

### Variables and measurement

The sample of variables included anthropometric indices and specific physical fitness tests. Anthropometric variables consisted of six variables: body height (BH), body mass (BM), body mass index (BMI), arm span (AS), triceps-skinfold (TrSF) and subscapular-skinfold (SsSF). The BH was measured by a stadiometer. BM was measured using a SECA weight scale, and the skinfolds using a Lange caliper. All measures were taken three times and a reliability analysis was performed (see below).

The specific physical fitness tests comprised four swimming tests (25m, 100m, 400m, and 4x50m), one specific explosive strength (power) test, and a specific dynamometric force test. The swimming tests included maximal swimming over 25m, 100m and 400m. In addition, we measured a specific anaerobic test consisting of swimming 50 m four times (4x50m). In this test a subject swam at their maximum exertion a 50 m distance four times, and between each trial there was a pause of 30 seconds. As a final result the average time of the four trials was used. To test specific explosive strength (power) we used a test involving a vertical one-arm body jump from the water (JUMP). The aim was to jump from the water as high as possible. The jump started from a vertical swimming position; a subject quickly rose out of the water and lifted one (preferred) arm as high as possible before running out of upward momentum.

The test was performed three times and after the reliability analysis the best result achieved was kept for further analysis. The semi-tethered dynamometric test (DYN) consisted of maximum intensity swimming with a fastened elastic line fixed to a special belt. Swimming force was recorded with the use of a tensiometric Baseline Evaluation Instruments dynamometer (Fabrication Enterprises, Inc; NY, USA) coupled to amplifier and PC software. The subjects were instructed to swim as hard as possible and to achieve the maximal possible dragging force while swimming. The maximal force achieved was retained as the result from each subject. First day of testing we have measured anthropometrics, 25 meters, 100 meters, and JUMP. The second day of testing 400 meters of swimming and DYN was performed. Third day the subjects were tested on 4x50m.

### Analysis

Reliability analysis for the anthropometric variables JUMP and DYN was performed by calculating Cronbach’s Alpha coefficient (Alpha), and a coefficient of variation (CV). Following Kolmogorov Smirnov’s test of the normality of distribution, we calculated means and standard deviations for each variable. Analysis of variance (ANOVA) with a post-hoc comparison was used to determine possible differences between the playing positions.

## Results

Reliability analysis showed high between-subject reliability for the anthropometric variables, with Alpha ranging from 0.81 (for SsSF) to 0.99 (for BH). The CV showed moderate-to-high within-subject variation and ranged from 0.09 (for SsSF) to 0.01 (for BH). Both specific fitness tests undertaken in the three trials showed moderate reliability, observed through the Alpha (0.78 and 0.81) and satisfactory CV (0.11 and 0.10 for DYN and JUMP, respectively (Table 1).

ANOVA found significant differences between the playing positions, but rarely with some post-hoc differences. However, there is an evident tendency for the Drivers to be the shortest of all, and a similar trend is evident for AS. The Centers are the heaviest and have the highest BMI of all players, while there are no significant differences in BM and BMI among the other positions. ANOVA identified the Centers as players with the largest skinfold thickness, with significant post-hoc differences for SsSF when the Centers were compared to the Goalkeepers and Wings. The significant differences were found between the playing positions in some swimming tests, and the Points achieved the best results in the 25m and 400m, with no significant post-hoc differences. The Centers also dominated in the JUMP, while the Goalkeepers achieved the lowest result for DYN. There were no significant ANOVA effects for 100m and 4x50m (Table 2).

## Discussion

Ferragut et al. (2011) recently studied the specific physical structure (i.e. anthropometric profile) of elite Spanish water polo players (aged 24 years on average) and reported 184, 187 and 192 cm as the average BH; 192, 192 and 203 cm as the average arm span for Wings, Points and Centers. It is therefore clear that the junior water polo players we studied herein are significantly taller and have a longer arm span than their older Spanish peers. An additional comparison shows that the senior Spanish Centers are on average 7 kg heavier and have approximately a 1 kg/m^2^ larger BMI than the juniors we studied. A similar trend of differences is evident for the Points, with the Spanish seniors being approximately 4 kg heavier and having a somewhat higher BMI than the juniors we studied. However, these differences are to be observed as related to advanced lean body mass and not body fat because of the smaller values of the skinfold measures among the Spanish senior water polo athletes in comparison to our juniors. Although the anthropometric differences between the Spanish seniors and Croatian junior water polo athletes can be interpreted differently, we have no doubt that the evident advantage of the Croatian juniors in their body length dimensions should be understood as a result of the overall trend in Croatian water polo. For example, two years ago M. Lozovina et al. (2009) presented anthropometric indices for senior Croatian water polo players. When compared to the anthropometric characteristics of the Spanish players (Ferragut, et al., 2011) it is clear that Croatian players are generally taller and heavier than their Spanish colleagues. Therefore, we can emphasize that the junior players we studied reflect an overall trend in Croatian water polo which favors tall players. The clear advancement of the Croatian seniors (M. Lozovina, et al., 2009) over the juniors we studied here should be explained by emphasizing two issues. First, although the juniors presented in this study are in the last phase of their growth and development, a minimal improvement of their BH should be expected even in the following year or two. Second, a player’s tallness will be surely be favored in the ensuing sport selection process which is known to be particularly strict between junior and senior ages (Jelicic et al., 2002). Therefore, it is expected that only players with an advanced BH will continue with active water polo in later (senior) ages.

We found numerous differences between the playing positions in their anthropometric features. The Centers, Points and Goalkeepers are the tallest, followed by the Wings, while the Drivers should be considered the shortest. This is naturally followed by AS an another measure of body length (i.e. longitudinal body dimension (Jelicic, et al., 2002). Very similar findings regarding differences in body length dimensions have already been found in the previously discussed investigations of Spanish (Ferragut, et al., 2011) and Croatian (M. Lozovina, et al., 2009) senior high-level players. We have no doubt that the background to such a situation should be found in the position-specific orientation process in water polo. In short, water polo is organized through defense and offense, and the characteristic game-tasks are well organized. The Points and Centers must be able to cover as much distance as possible while swimming in an upward position. Such game tasks directly favor taller players, chiefly because of their longer arms. This allows them to reach higher and further for the ball. In addition, body length allows them to keep a distance from an opponent during the contact game, which is most frequent between the opposed Points and Centers. The need for advanced body lengths among goalkeepers has already been studied with regard to other team sports (Wong et al., 2009). In short, it is obvious that this anthropometric characteristic allows them to cover the wider space of the goal and hence to defend the net more successfully.

Because of the constant contact during the game, Centers are known to be the largest of all players in terms of body length and body mass. Therefore, it was not surprising that, although similar to the Points and Goalkeepers in BH, the Centers are the heaviest and have the highest BMI of all five playing positions. Apparently, their increased BM and BMI are partially but not entirely related to increased body fat (i.e. Centers have higher skinfolds than the Goalkeepers and Wings, but there is no significant difference in any of the body fat measures between the Centers, Points and Drivers). This is in line with previous findings where authors discussed the clear need for a Center’s morphological-anthropometric dominance in terms of advanced BM, especially against rival Points (M. Lozovina, et al., 2009). More precisely, these two playing-positions are direct opponents (i.e. the Point guards the offensive Center) and if a Center wants to be effective in his/her offensive tasks, he/she must be physically superior to the defensive player guarding him (her).

Although previous studies rarely studied water polo goalkeepers with regard to their anthropometric status, the results of the Goalkeepers’ anthropometric variables did not surprise us. Most particularly, they are slightly, although not significantly dominant in AS, and have the lowest BMI of all players. Such an anthropometric profile allows them to cover the net efficiently (because of their large arm span) and to change position quickly (because of their low BMI). Since the official rules of water polo protect Goalkeepers from the contact-game, their low BMI is clearly a function of their agile movement and quick positioning in front of the goal with regard to offensive actions and his/her team’s defensive tactics.

The importance of the specific physical fitness profile of different playing positions is already recognized in team sports (Ben Abdelkrim et al., 2010; Markovic and Mikulic, 2011; Pyne et al., 2006), but such studies are evidently scarce in water polo, especially among junior players. Therefore, the results of the specific physical fitness tests we presented above are hardly comparable to previous findings. Although the playing positions did not differ significantly in the lactate capacity (4x50m) and 100m swimming results, the swimming performance measured by swimming 25m (ATPCP capacity), and 400m (aerobic capacity) revealed the Points to be the best swimmers. According to previous studies, the background to such findings should be identified through anthropometric profiles. In a recent study where authors identified the optimal morphological/anthropometric characteristics of young competitive swimmers, Sekulic et al. (2007) defined the linear influence of BH on short-distance swimming (SDS) performance (i.e. taller swimmers performed better). At the same time, a non-linear relationship between BM and SDS was found. Most particularly, BM positively influenced SDS, but only up to average BM results. With greater BM values (above the average results for the studied sample), a negative influence of BM on SDS was evidenced. Both of these findings support our results whereby the Points performed better in SDS (25m). In short, the Points are the tallest of all players, but at the same time they do not have the highest BM (i.e. the Centers are heavier). Such an anthropometric profile allows them to utilize the length of their longer extremities (i.e. longer arms and legs) and therefore to execute fewer arm strokes over the same distance (Potdevin et al., 2006). Altogether, advanced BH with an adequate BM allows them to achieve a higher moment of force (MF) in a single stroke due to the law of levers (*MF = F × b*). In this particular case, “F” is the force applied during a single arm stroke, and “b” is the distance between a single joint and connection point of the active muscles on the bone (lever). Naturally, “a” increases with BH, which is followed by an increase in MF, all allowing one to swim faster. Of course, all of the above can only be expected if BH is followed by an adequate BM; in other words, muscle mass as a generator of force. The Points also performed best in the 400m test, which should be identified as an aerobic-capacity performance (approximately 6 minutes of continuous work). Although not investigated systematically, we must note it would be hard to expect such a performance over 400m among senior-age Points. In short and as discussed, the BM of the Points increases during the following period (from junior to senior age), which is hardly followed by similar dynamics of an increase in BH (i.e. lengths of body segments).

In water polo, the performance of a vertical water-jump is particularly important and is evident in each situation where a player must reach vertically out of the water as part of shooting, passing and/or blocking an opponent. Jumping performances are probably even more crucial for goalkeepers since their playing efficacy directly relates to frequent jumping (Platanou, 2005). The results of our subjects do not evidently differ from the results of the same tests presented for Greek Premier League players (Platanou, 2006). Significant differences among the playing positions showed the dominance of the Points in this test. One could argue that this mostly relates to anthropometric status (the greater BH of the Points, see above) and it is evidently correct. In short, as we additionally calculated, the differences among playing positions in their relative jumping capacity (i.e. when absolute jumping performance is divided by BH) are not significant (F = 1.08; p = 0.387). However, like in other team sports, in water polo a jumping performance should be observed as “absolute” and not “relative” (Platanou, 2006).

Consequently, notwithstanding the fact that the anthropometric and not the physiological background led to differences in JUMP among the playing positions, the Points should be judged as the most successful of all players in this specific physical fitness test.

Although used among swimmers (Secchi et al., 2010), DYN test results are rarely investigated among water polo players. Finding the lowest values of the passive drag force production among goalkeepers is not surprising, and once again such results are to be observed as directly influenced by anthropometric characteristics. As discussed, the Goalkeepers have the lowest BMI of all players, with relatively long extremities (a large arm span). Such an anthropometric profile logically does not allow them to produce a high drag force during semi-tethered swimming but, in contrast, it assures fast and agile movements which are vital prerequisites of efficient goalkeeping. Therefore, given their game duties their poor achievement in DYN should not be seen as some kind of handicap. The highest values for DYN are achieved by the Centers, followed by the Points, which is also logical mainly because of their superior body build relative to the other players. However, from our point of view, the relatively small differences between the Centers and Points in this particular test defines this performance as a certain weak point of the Centers’ physical capacity relative to their direct opponents – the Points. It is known that the production of force depends directly on lean body mass (F = m x a) and partially on the length of body segments where the movements are completed (i.e. arms and legs in this case; see above where we discussed the influence of BH on swimming performance). Since the Centers are far heavier than the Points (8kg on average), and similar to them in body fat measures, BH and AS, this clearly implies the possibility of the far more advanced production of dynamometric force among the Centers in comparison to the Points. Knowing the previously discussed differences in swimming results (i.e. the Points’ dominance in swimming performance over short and long distances), but also based on the authors’ professional experience with water polo, we believe the main reason for such inconsistency (i.e. by all means the Centers should dominate in DYN) is to be found in the relatively poor swimming technique of the Centers. It is most likely that for that reason their superior morphological capacities are not properly exploited during the DYN testing.

## Conclusion

The position-specific anthropometric profiles of junior water polo players are in line with previously reported results for senior-age players. However, a comparison of our results with those of senior-age players showed there is a real possibility that in the following period (between junior and senior ages) the sport-selection process will favor tall players.

Since we studied a representative sample of junior water polo players, which included one of the best national junior teams of the world in the 2009/10 season, the data presented for the different playing positions should be observed as numerical norms of the anthropometrical measures we studied. The same can be said for the specific physical fitness tests we studied here which should be used in two separate ways. First, they should be used as orientation values to allow coaches to compare the results their players achieve with the results presented here and to emphasize the need for specific training. This will allow coaches to design appropriate training programs aimed at improving the specific physical abilities of water polo players in different positions, while keeping their anthropometric features in mind. Finally, by applying the results presented here water polo coaches will be able to place their players in the most appropriate playing positions according to their physical capacities and anthropometric characteristics.

## Figures and Tables

**Table 1:** Descriptive statistics (Means – mean; SD – standard deviation; Min – minimum; Max – maximum), and reliability analysis (ALPHA – Cronbach Alpha; CV – coefficient of variation) of the general anthropometric and specific physical fitness tests

	Mean	SD	Min	Max	ALPHA	CV
BH (cm)	186.92	6.31	173.00	204.60	0.99	0.01
BM (kg)	84.31	9.46	63.00	112.00	0.98	0.02
AS (cm)	194.99	7.60	177.50	212.50	0.95	0.07
TrSF (mm)	11.27	3.32	6.00	19.20	0.88	0.07
SsSF (mm)	12.57	3.37	6.80	22.60	0.81	0.09
JUMP (cm)	145.24	6.71	129.00	160.00	0.81	0.10
DYN (kg)	34.23	16.33	13.50	78.00	0.78	0.11

BH - body height, BM - body mass, AS - arm span, TrSF – triceps skinfold; SsSF – subscapular skinfold; JUMP – vertical one-arm body jump from the water; DYN – semi tethered swimming dynamometric test

**Table 2:** Analysis of variance (F – F test value;^*^ denotes significant F test value) between playing positions (Means ± Standard deviation), with post-hoc differences using the Scheffe test

	Points Mean±SD	Centers Mean±SD	Goalkeepers Mean±SD	Wings Mean±SD	Drivers Mean±SD	F
BH (cm)	189.97±6.17	189.67±5.56	189.68±6.78	187.14±2.73	183±5.32	3.22^*^
BM (kg)	87.85±7.06	95.85±8.85 ^G,W,D^	82.04±8.38 ^C^	83.32±3.92 ^C^	80.35±7.53 ^C^	9.78^*^
BMI (kg/m^2^)	24.37±1.9 ^C^	26.62±1.9 ^P, G,W,D^	22.78±1.8 ^C^	23.79±0.92 ^C^	24.01±2.2 ^C^	9.75^*^
AS (cm)	197.67±7.85	198.31±6.24	198.98±7.26	193.6±3.46	190.7±7.65	3.24^*^
TrSF (mm)	11.14±3.08	11.79±4.08	9.93±2.56	10.63±3.12	12.04±3.52	1.04
SsSF (mm)	13.07±3.52	15.52±3.42 ^G, W^	10.89±2.76 ^C^	10.29±1.78 ^C^	12.35±2.97	6.29^*^
25m (s)	12.95±0.66	13.4±0.49	-	13.1±0.92	13.11±0.55	2.55^*^
400m (s)	271.09±48.65	301.78±14.78	-	298.51±23.82	283.31±45.93	2.75^*^
100m (s)	64±6.93	63.32±3.4	64.37±6.73	61.85±4.42	63.86±8.38	0.25
4x50 (s)	31.91±3.67	30.81±1.57	-	30.87±2.24	31.8±4.03	0.51
JUMP (cm)	148.3±7.14	143.73±5.81	144.05±6.48	143.84±5.22	142.45±5.34	2.79^*^
DYN (kg)	36.71±19.46 ^G^	37.29±18.3 ^G^	28.31±10.41 ^P,C,W,D^	35.17±12.91 ^G^	35.81±16.3 ^G^	2.77

BH - body height, BM - body mass, BMI - body mass index; AS - arm span, TrSF – triceps skinfold; SsSF – subscapular skinfold; 25m – swimming 25 meters; 100m – swimming 100 meters; 400m – swimming 400 meters; 4x50 – average time of 4-time-50 meters swimming with 30 seconds rest; JUMP – vertical one-arm body jump from the water; DYN – semi tethered swimming dynamometric test;

P denotes significant post-hoc differences when compared to Points;

C denotes significant post-hoc differences when compared to Centers;

G denotes significant post-hoc differences when compared to Goalkeepers;

W denotes significant post-hoc differences when compared to Wings;

D denotes significant post-hoc differences when compared to Drivers
